# Novel approaches to the study of viscosity discrimination in rodents

**DOI:** 10.1038/s41598-022-20441-y

**Published:** 2022-09-30

**Authors:** Chihiro Nakatomi, Noritaka Sako, Yuichi Miyamura, Seiwa Horie, Takemi Shikayama, Aoi Morii, Mako Naniwa, Chia-Chien Hsu, Kentaro Ono

**Affiliations:** 1 Division of Physiology, Kyushu Dental University, Fukuoka, 803-8580 Japan; 2 Department of Oral Physiology, Asahi University School of Dentistry, Gifu, 501-0296 Japan; 3 Division of Orofacial Functions and Orthodontics, Kyushu Dental University, Fukuoka, 803-8580 Japan

**Keywords:** Neuroscience, Physiology

## Abstract

Texture has enormous effects on food preferences. The materials used to study texture discrimination also have tastes that experimental animal can detect; therefore, such studies must be designed to exclude taste differences. In this study, to minimize the effects of material tastes, we utilized high- and low-viscosity forms of carboxymethyl cellulose (CMC-H and CMC-L, respectively) at the same concentrations (0.1–3%) for viscosity discrimination tests in rats. In two-bottle preference tests of water and CMC, rats avoided CMC-H solutions above 1% (63 mPa·s) but did not avoid less viscous CMC-L solutions with equivalent taste magnitudes, suggesting that rats spontaneously avoided high viscosity. To evaluate low-viscosity discrimination, we performed conditioned aversion tests to 0.1% CMC, which initially showed a comparable preference ratio to water in the two-bottle preference tests. Conditioning with 0.1% CMC-L (1.5 mPa·s) did not induce aversion to 0.1% CMC-L or CMC-H. However, rats acquired a conditioned aversion to 0.1% CMC-H (3.6 mPa·s) even after latent inhibition to CMC taste by pre-exposure to 0.1% CMC-L. These results suggest that rats can discriminate considerably low viscosity independent of CMC taste. This novel approach for viscosity discrimination can be used to investigate the mechanisms of texture perception in mammals.

## Introduction

Food texture perception plays important roles in mastication and swallowing^1–5^. Food textures were classified by Szczesniak in 1963 in terms of mechanical, geometrical, and other characteristics (such as hardness and viscosity)^6^. During mastication and swallowing, food textures are physically received on the surface of the mouth, oral cavity, and pharynx by mechanoreceptors (mainly Krause and Meisner corpuscles and Merkel cells)^7,8^. The mechanical activation of the peripheral afferents is conducted to the somatosensory cortex and amygdala through the thalamus and is perceived as tactile quality and preference/aversion. However, the neurological mechanisms of texture sensation and perception have not been studied in detail in mammals.

The lack of progress in the research field has been due to methodological problems in experiments for food texture perception. Although experimenters try to select tasteless additive materials to add texture properties, the selected additive materials seem to have some taste for experimental animals (for example, starch and cellulose)^9,10^. A pioneering study for viscosity discrimination reported that rats can discriminate high viscosity using conditioned aversion tests with different thickeners^11^. However, because common thickeners have tastes, it is difficult to strictly interpret whether the changes in behaviors are due to textures or tastes. Therefore, an animal experimental assay must be developed to analyze texture perception without the effects of taste.

In the present study, to minimize the taste effects of thickeners, we used low- and high-viscosity forms of carboxymethyl cellulose (CMC-L and CMC-H, respectively); the only difference between CMC-L and CMC-H is the degree of polymerization. Hence, both solutions have the same taste at any given concentration. Conditioned aversion is an associative learning wherein a conditioned stimulus (CS), such as a taste or physical property of foods, is paired with malaise (unconditioned stimulus, US)^11–15^. Many studies have shown that pre-exposure to a taste without US reduce the acquisition of taste aversion in rats^16–22^, referred to as latent inhibition (LI). LI is sensitive to the properties of the pre-exposure stimulus, including its intensity (concentration), frequency, and interval^16–20^. In this study, we carefully planned the pre-exposure procedure to induce LI only for the taste of CMC in the conditioned viscosity aversion tests.

## Methods

### Viscous fluids

CMC-L and CMC-H sodium salt (Sigma–Aldrich, St. Louis, MO) were added at 0.1, 0.3, 1.0, and 3.0% to distilled water or 0.08% saccharin (Wako, Osaka, Japan)-containing water. Xanthan gum (Tokyo Chemical Industry Co., Ltd., Tokyo, Japan) and a commercial thickener Tsururinko-Quickly® (dextrin, 30% xanthan gum, calcium lactate and trisodium citrate) (Clinico Co., Ltd, Tokyo, Japan) were added at 1.5 and 3.0% in water. These thickener-containing fluids were prepared 1 day prior to usage. The viscosity of fluids was measured with a round vibrational viscometer (Viscomate VM-10A, CBC Materials Co., Ltd., Japan) with a probe that oscillates at 500 Hz in a sample volume of 10 ml inside a purpose-made glass cup. Each fluid was prepared 3 times and measured. The viscosity of distilled water was 0.93 mPa·s^41^.

### Animals

Male Wistar rats (n = 140, 230–250 g; CLEA Japan, Inc., Tokyo, Japan) were used in this study. Since male rats have been reported to show a stronger response than female rats in the conditioned aversion test^42^, we selected male rats for the animal experiments. The rats were housed in a clear cage under specific-pathogen-free conditions and maintained on a 12:12 h light: dark cycle in a temperature- and humidity-controlled environment (21–23 °C and 40–60%, respectively) with food pellets and water provided ad libitum except during experiments. All animal behavioral experiments were approved by the Animal Experiment Committee of Kyushu Dental University (Approval number: 19-018) and conducted in accordance with the National Institutes of Health guidelines (Guide for the Care and Use of Laboratory Animals). The number of rats was based on the minimum required for statistical analysis. Electrophysiological experiments were conducted according to the “Guidelines for the Proper Conduct of Animal Experiments (Science Council of Japan; 2006)” and “The Animal Care Guidelines of Asahi University.” The test protocols were approved by “The Animal Care and Ethics Committee of Asahi University” (Approval Nos: 18-039, 19-013, 20-002, and 21-001). This study conformed with the ARRIVE (Animal Research: Reporting In Vivo Experiments) guidelines for animal studies.

Glass jar containers were used during the animal experiments (Fig. 2a). The round container filled with fluid was placed on a plastic duckboard on wood chips in the bottom of the cage (Fig. 2a). The container was covered by a stainless cap with a hole to allow a rat access to fluid. The duckboard and container cap were needed to prevent the fluid from becoming contaminated with wood chips from the cage floor. Rats were acclimatized to the experimental conditions for a week before experiments. All experimental sessions were conducted at a constant time (10:00–12:00) following water and food deprivation for 12 h (22:00–10:00). The rats were allowed free access to both containers during the test periods.

### Two-bottle preference tests for viscous fluids

Two pre-weighed containers that were filled with thickener-containing solution or water were placed in a cage for 60 min (Fig. 2b). The position of the containers was switched every 15 min. A rat was used for 3–4 different tests at 3- to 4-day intervals and received test solution 12 h prior to the water and food deprivations for each experimental session to minimize neophobia to the test solution (n = 25). Fluid intake in a container was measured by subtracting the posttest weight of a container from the pretest weight and then standardized with body weight. The preference ratio was calculated by dividing the intake of the test solution by the total intake of both solutions.

### Conditioned aversion tests after the pre-exposures to viscous fluids

Rats were pre-exposed to viscous fluid for various periods before aversion conditioning to minimize the effect of the taste of the viscous fluids. During the pre-exposure periods, two containers were placed in the cage, one filled with water and the other with viscous fluids. Rats were allowed free access to viscous fluids and water.

In the long-term pre-exposure experiments, rats were pre-exposed to viscous fluids, 0.1% CMC-L or CMC-H, for 8 days, followed by water and food deprivation for 12 h. On the conditioning day (day 0), the rats were served viscous fluids, indicated by an arrow in each graph, as CS for 10 min. After confirming sufficient fluid intake, the conditioned groups were intraperitoneally administered lithium chloride (LiCl) (0.15 M in saline, 2 ml/100 g body weight; Nakarai Tesque, Kyoto, Japan) to induce gastrointestinal malaise. The vehicle group was administered the same volume of saline. Water and food were returned to the cage 1 h after the procedures. After conditioning, the rats were presented only with distilled water (DW). On the 3 days after the conditioning trial, the preference ratio for the test fluids was measured by using a similar procedure described above. Rats were deprived of water and food for 12 h before the preference tests.

In the short-term pre-exposure experiments, rats were pre-exposed to test fluids for 12 h and deprived of water and food for 12 h. Subsequently, baseline values of the preference ratio for test fluids were measured. After the measurement of baseline values, rats were divided into conditioned and vehicle groups (n = 5 for each group) and were presented only with DW for 4 days. On the conditioning day (day 0), rats were trained to avoid various viscous fluids by a procedure similar to the one described above. Three days after the conditioning trial, the preference ratio for the test fluids was measured. For 3% Tsururinko®, the preference ratio was measured for 28 days.

In the experiments without pre-exposure, conditioned aversion tests were carried out without any pre-exposure treatments.

### Electrophysiological experiments

The rats were deeply anesthetized with an intraperitoneal injection of a combination of anesthesia compounded with 0.375 mg/kg medetomidine, 2.0 mg/kg midazolam and 2.5 mg/kg butorphanol. After surgical level of anesthesia was achieved, each animal was tracheotomized and secured by a head holder. The chorda tympani nerve was exposed by the lateral approach, cut near its entrance into the tympanic bulla, and dissected free from the underlying tissues. The nerve was desheathed and placed on a platinum wire recording electrode. An indifferent electrode was attached to nearby tissues. The activity of the whole nerve was amplified, displayed on an oscilloscope, and monitored with an audio amplifier. The amplified signals were passed through an integrator with a time constant of 0.3 s, and they were also monitored on a PowerLab® (ADInstruments, New Zealand). The test solutions were 0.1 M NH_4_Cl, 0.1% CMC-L, 0.1% CMC-H, 1% CMC-L, and 1% CMC-H_._ Each stimulus (1 ml) was applied to the anterior dorsal tongue for 20 s, followed by a distilled rinse for at least 45 s. The magnitude of each response to stimuli was adopted as the medium value of integrated response from the baseline from 8 to 18 s after onset of stimulation by using PowerLab®. The response to each stimulus was expressed as the relative magnitudes of responses when the magnitude of response to 0.1 M NH_4_Cl was taken as the standard (0.1 M NH_4_Cl = 1.0).

### Statistical analysis

Statistical analyses were performed using GraphPad Prism software (GraphPad Prism® 6 for Windows, Version 6.07). Data are presented as the mean ± standard error of the mean, and n represents the number of rats tested. An unpaired two-tailed Student’s *t* test was used to compare differences between two different groups or experimental days. The Sidak’s post hoc test following two-way analysis of variance (ANOVA) was used to analyze CMC dose changes between the low- and high-viscosity types or xanthan gum dose changes between Tsururinko® and xanthan gum only solutions. Significance was accepted at *P* < 0.05.

## Results

### Viscosity and taste of CMC solutions

**Figure 1:**
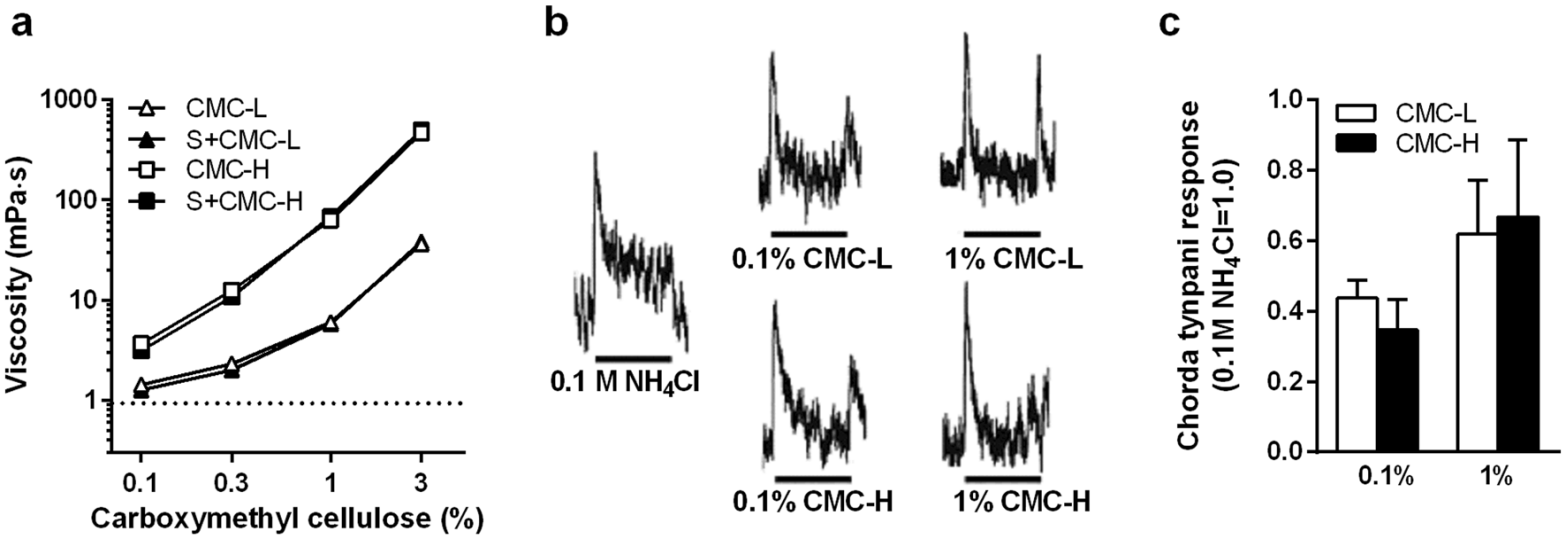
Mean viscosities and integrated whole chorda tympani responses to carboxymethyl cellulose (CMC) of low- or high-viscosity type. Mean ± standard error of the mean. CMC-L, low-viscosity CMC fluid; S + CMC-L, saccharin-containing CMC-L fluid; CMC-H, high-viscosity CMC fluid; S + CMC-H, saccharin-containing CMC-H fluid. (**a**) Mean viscosities of CMC-containing water with or without 0.08% saccharin (3 measurements). Both x- and y-axes are logarithmic scales. (**b**) Representative integrated whole chorda tympani. Viscous fluid stimuli were applied for 20 s as indicated by underlines. (**c**) Relative magnitude of responses of the chorda tympani to viscous fluid stimuli (each bar, n = 5). The response magnitude is expressed when the magnitude of the response to 0.1 M ammonium chloride (NH_4_Cl) is taken as 1.0.

The viscosities of CMC-H were higher than those of CMC-L at the same concentration (0.1–3%; Fig. 1a). The addition of saccharin did not change the viscosity of CMC (Fig. 1a). Since an early study reported that rats can detect a cellulose taste^9^, we investigated the neuronal response of the taste nerve (the chorda tympani) in response to CMC solutions. As shown in Fig. 1b, the rat chorda tympani responded to 0.1% and 1% CMC-L and CMC-H. The response patterns to viscous fluids were relatively unstable compared with a control solution (ammonium chloride, NH_4_Cl). Sharp responses occurred when wash water was dropped after CMC applications but not after NH_4_Cl application (Fig. 1b). The response patterns and intensity were almost the same between CMC-L and CMC-H at the same concentrations (Fig. 1b,c). The results suggested that CMC-L and CMC-H had similar tastes. The response to 1% CMC-L and CMC-H was greater than that to 0.1% CMC-L and CMC-H, although there were no statistically significant differences (Fig. 1c).

### Preferences for CMC solutions

**Figure 2:**
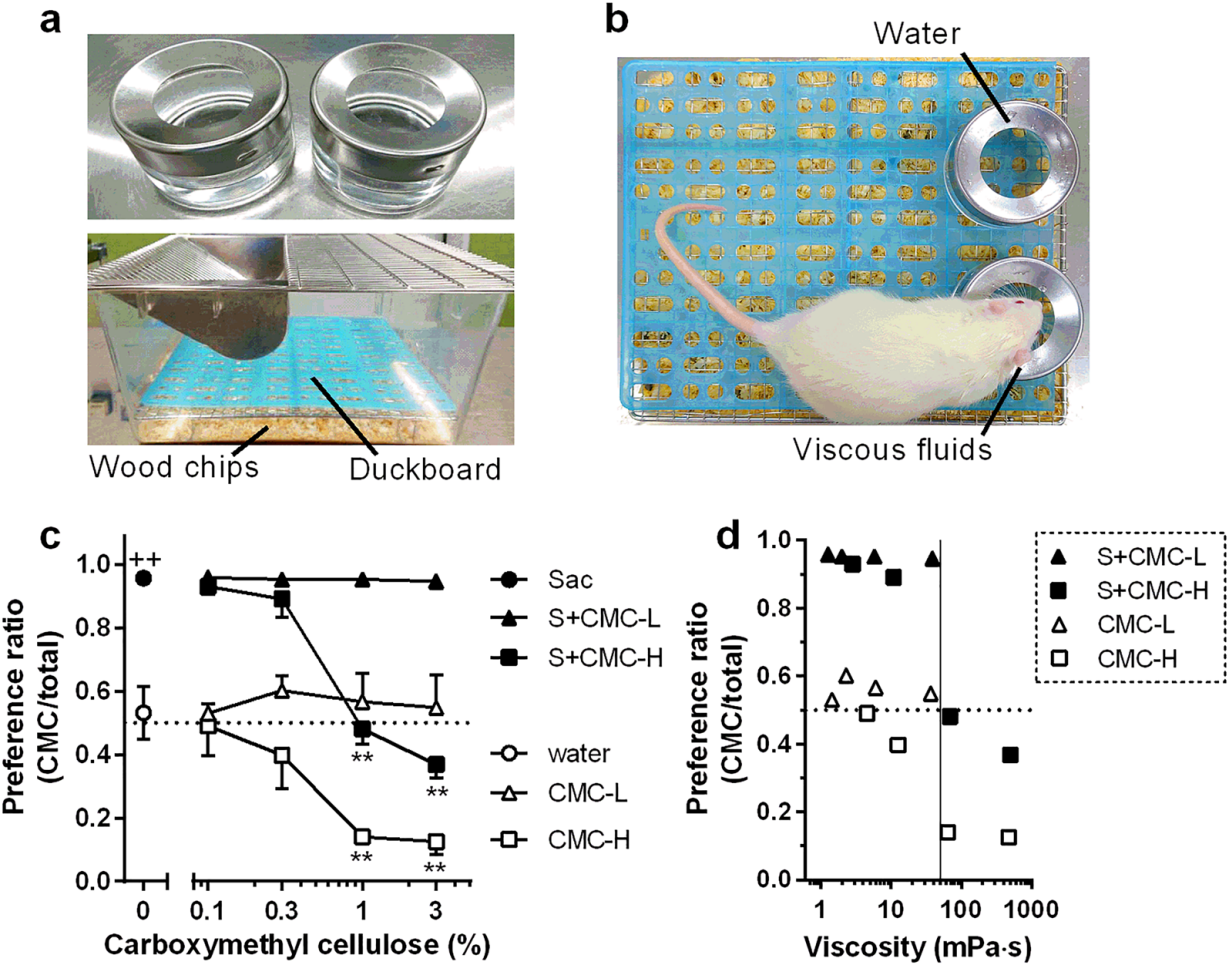
Preference for carboxymethyl cellulose (CMC) in rats. CMC-L, low-viscosity CMC fluid; S + CMC-L, saccharin-containing CMC-L fluid; CMC-H, high-viscosity CMC fluid; S + CMC-H, saccharin-containing CMC-H fluid. Mean ± standard error of the mean. (**a**) Photographs of glass jar containers and a cage used in the experiments. (**b**) Photographs of two-bottle preference tests. (**c**) Preference ratio of CMC-containing fluids (each point, n = 5). **, *P* < 0.01 against CMC-L or S + CMC-L fluid in Sidak’s post hoc test following two-way ANOVA test. ^++^, *P* < 0.01 against water by unpaired two-tailed Student’s *t* test. (**d**) Relationship of viscosity and preference ratio of CMC-containing fluids.

In two-bottle preference tests of CMC vs. plain water, preferences for CMC-H above 1% were significantly lower than those for CMC-L at the same concentration (*P* = 0.0011 and *P* = 0.0013 against 1% CMC-L and 3% CMC-H, respectively, in Sidak’s post hoc test following two-way ANOVA; Fig. 2c). To examine the effect of an obvious taste on viscous fluid intake, we added saccharin (a calorie-free sweetener) at 0.08% into CMC solutions. Expectedly, saccharin-containing water showed a higher preference ratio than plain water (*P* = 0.0009, in unpaired two-tailed Student’s *t* test; Fig. 2c). Despite the high preference for saccharin-containing CMC, preferences for saccharin-containing CMC-H at 1% and 3% were significantly lower than those for saccharin-containing CMC-L at the same concentration (*P* = 0.0018 and *P* = 0.002 against 1% CMC-L and 3% CMC-H, respectively, in Sidak’s post hoc test following two-way ANOVA; Fig. 2c). Figure 2d shows plots of the mean preference ratio for CMC against viscosity. The preference ratio for CMC solutions with and without saccharin decreased drastically at viscosities above 63 mPa·s (Fig. 2d).

### Discrimination of low viscosity without or with LI

**Figure 3:**
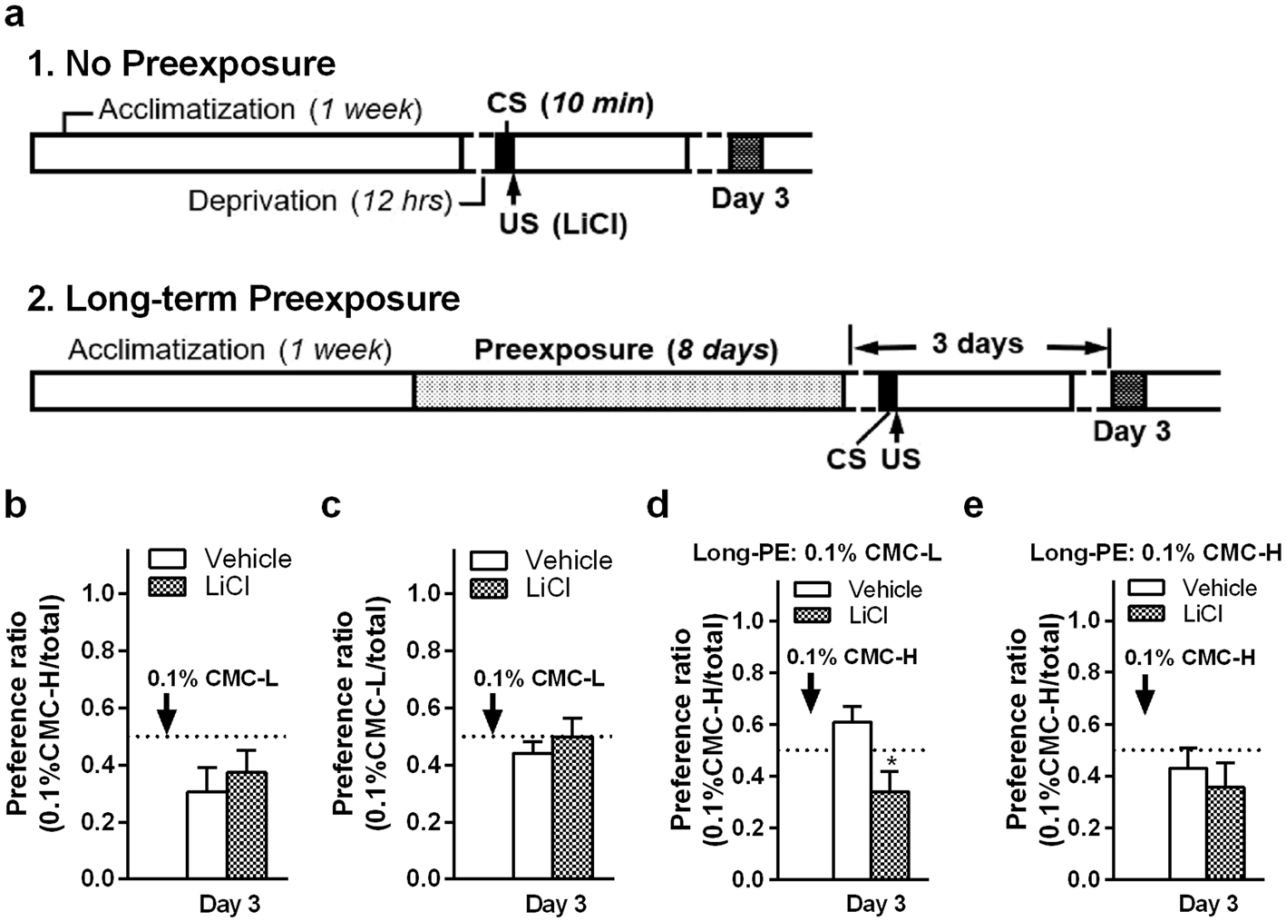
Conditioned aversion tests to 0.1% low- or high-viscosity carboxymethyl cellulose (CMC)-containing fluids 3 days after lithium chloride (LiCl) injection (unconditioned stimulus [US]) following ingestion of a viscous fluid (conditioned stimulus [CS], indicated by an arrow in each graph). CMC-L, low-viscosity CMC fluid; CMC-H, high-viscosity CMC fluid. Mean ± standard error of the mean. (**a**) Either no pre-exposure or long-term (8 days) pre-exposure to CMC solutions was carried out before conditioning. A pre-exposure procedure was performed to acquire safe learning of the CMC taste in rats. (**b**,**c**) Without pre-exposure, preference ratio in water and 0.1% CMC-H (**b**) or 0.1% CMC-L (**c**) after LiCl injection following ingestion of 0.1% CMC-L as CS (each group, n = 5). (**d**,**e**) Following long pre-exposure to 0.1% CMC-L (**d**) or 0.1% CMC-H (**e**), preference ratio in water and 0.1% CMC-H after LiCl injection following ingestion of 0.1% CMC-H as CS (each group, n = 5). *, *P* < 0.05 against vehicle on the same tested day by unpaired two-tailed Student’s *t* test. PE, pre-exposure.

To explore low-viscosity discrimination, we conducted conditioned viscosity aversion tests using 0.1% CMC-L and 0.1% CMC-H, which had the same taste and a small difference in viscosity (Fig. 1a). Previous studies have suggested that total pre-exposure time and short interval from the pre-exposure to the test are important factors to determine LI magnitude^16–19^. Therefore, we carried out two different procedures as follows: the first procedure was without pre-exposure before conditioning, and the second procedure was with long-term pre-exposure with a minimum pre-exposure–test interval to induce LI for CMC, as summarized in Fig. 3a.

We measured the preference for 0.1% CMC-L and CMC-H after conditioning to 0.1% CMC-L, according to the first procedure. Although 0.1% CMC-L and CMC-H had the same taste, conditioning to 0.1% CMC-L did not change the preference for 0.1% CMC-H (Fig. 3b). Furthermore, the same conditioning did not change the preference for the same 0.1% CMC-L (Fig. 3c), suggesting that the viscosity and taste of 0.1% CMC-L were too weak to induce aversion learning. According to the second procedure, to examine the viscosity discrimination of 0.1% CMC-H without any taste effect, we tried to induce LI by long-term pre-exposure to 0.1% CMC-L before conditioning (Fig. 3d). Importantly, even after long-term pre-exposure to 0.1% CMC-L, rats acquired conditioned aversion to 0.1% CMC-H (*P* = 0.023 in an unpaired two-tailed Student’s *t* test; Fig. 3d). On the other hand, following long-term pre-exposure to 0.1% CMC-H, rats did not acquire conditioned aversion to 0.1% CMC-H (Fig. 3e). The result indicates that LI for low viscosity is induced by the 8-day pre-exposure period and the 3-day pre-exposure–test interval.

### Discrimination of low viscosity with short-term pre-exposure to CMC solutions

**Figure 4:**
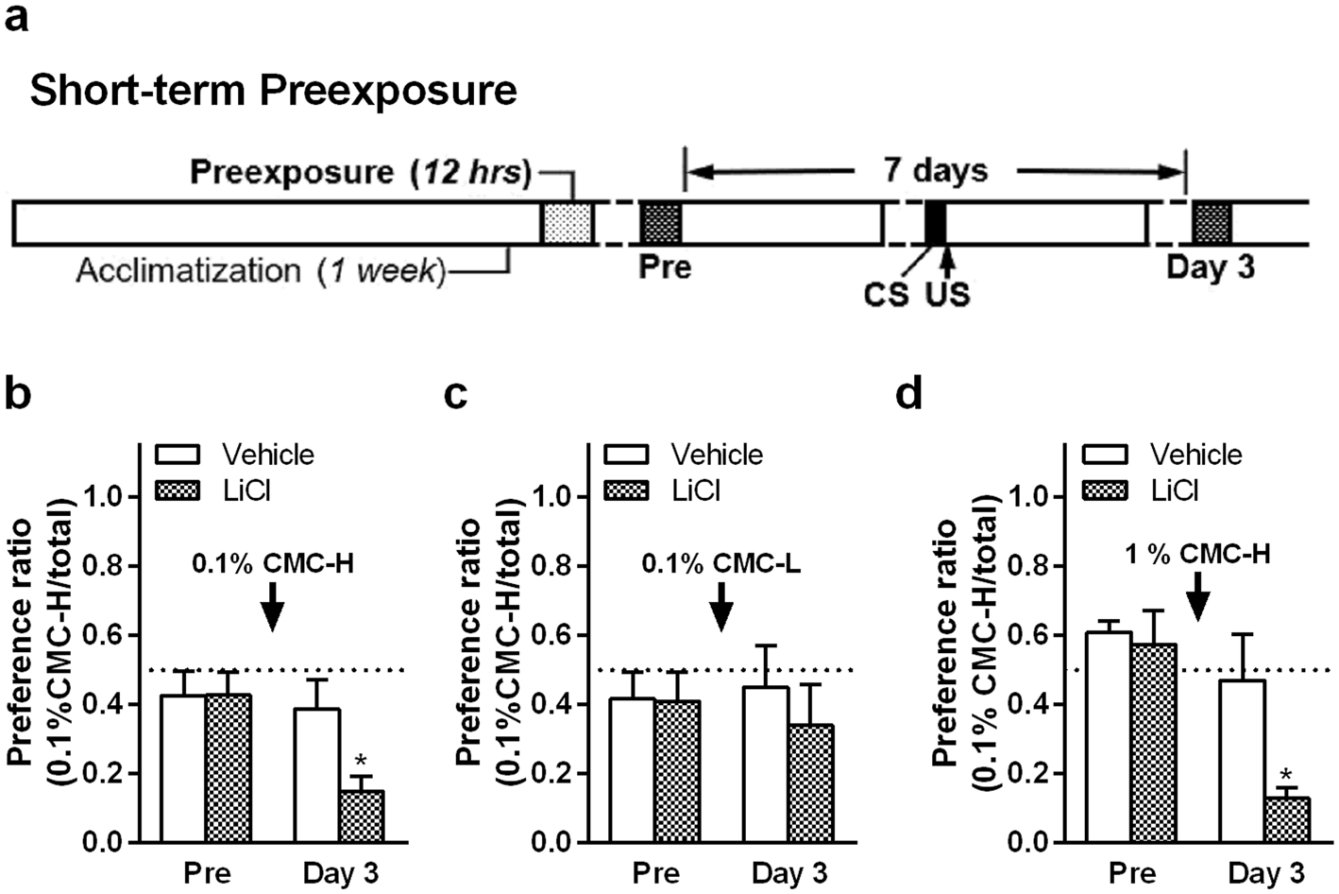
Conditioned aversion tests to 0.1% low- or high-viscosity carboxymethyl cellulose (CMC)-containing fluids 3 days after lithium chloride (LiCl) injection (unconditioned stimulus [US]) following ingestion of a viscous fluid (conditioned stimulus [CS], indicated by an arrow in each graph). CMC-L, low-viscosity CMC fluid; CMC-H, high-viscosity CMC fluid. Mean ± standard error of the mean. (**a**) Short-term pre-exposure (approximately half a day) to CMC solutions was carried out before conditioning. A pre-exposure procedure was performed to acquire safe learning of the CMC taste in rats. Following a short pre-exposure to 0.1% CMC-H with baseline value measurements, the preference ratio in water and 0.1% CMC-H after LiCl injection following ingestion of 0.1% CMC-H (**b**) or 0.1% CMC-L (**c**) or 1% CMC-H (**d**) as CS (each group, n = 5). *, *P* < 0.05 against vehicle on the same tested day by unpaired two-tailed Student’s *t* test.

Some early studies have reported that a single pre-exposure to a taste induces LI^18,19,21^, whereas a short-term pre-exposure to 30% Polycose, which has a viscosity of 4.0 mPa·s, has been reported to fail to induce LI^23–27^. Therefore, we hypothesized that pre-exposure to a viscosity is more difficult to induce LI than to taste. To selectively induce LI only for tastes and not for viscosity, we carried out a short-term pre-exposure to thickeners before conditioning with a long pre-exposure–test interval (Fig. 4a). Baseline values were measured during the short-term pre-exposure. After the short-term pre-exposure to 0.1% CMC-H with a 7-day pre-exposure–test interval, rats acquired conditioned aversion to 0.1% CMC-H (*P* = 0.0354, unpaired two-tailed Student’s *t* test; Fig. 4b), different from the long-term pre-exposure with a 3-day pre-exposure–test interval (Fig. 3e). This result suggests that the LI for viscosity is not induced by 12 h pre-exposure with a 7-day pre-exposure–test interval. After the same short-term pre-exposure to 0.1% CMC-H, conditioning to 0.1% CMC-L did not induce aversion to 0.1% CMC-H (Fig. 4c). Interestingly, conditioning to a higher viscosity solution (1% CMC-H) induced aversion to 0.1% CMC-H (*P* = 0.0373, unpaired two-tailed Student’s *t* test; Fig. 4d).

### Discrimination of viscosity in the presence of a sweet taste

**Figure 5:**
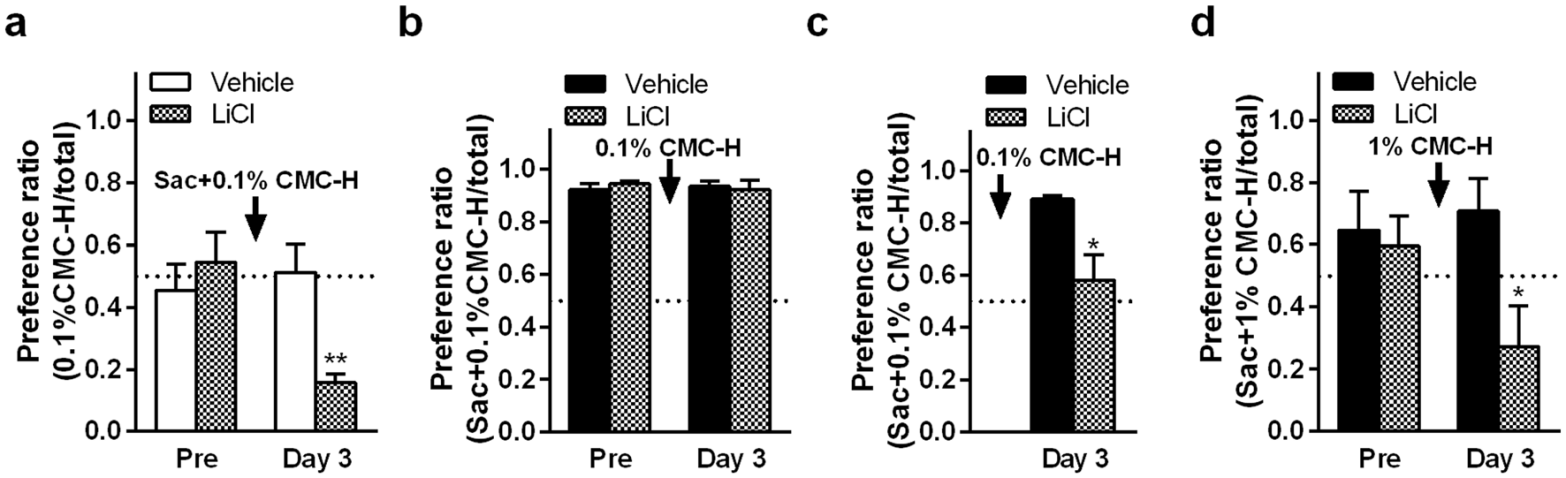
Effect of sweet taste on viscosity discrimination. Mean ± standard error of the mean. (**a**) Following short pre-exposure to high-viscosity carboxymethyl cellulose (CMC)-containing fluids (CMC-H) with baseline value measurements, preference ratio in water and 0.1% CMC-H after lithium chloride (LiCl) injection following ingestion of saccharin-containing 0.1% CMC-H fluid (Sac + 0.1% CMC-H) as conditioned stimulus (CS) (each group, n = 5). (**b**,**c**) Preference ratio in water and Sac + 0.1% CMC-H fluid after LiCl injection following ingestion of 0.1% CMC-H as CS with or without short pre-exposure, respectively (each group, n = 5). (**d**) Preference ratio in water and Sac + 1% CMC-H fluid after LiCl injection following ingestion of 1% CMC-H as CS with short pre-exposure (each group, n = 5). * and **, *P* < 0.05 and 0.01, respectively, against vehicle on the same tested day by unpaired two-tailed Student’s *t* test.

To examine viscosity discrimination in the presence of an obvious taste, we conducted conditioned viscosity aversion tests using the sweet taste saccharin-containing CMC after short-term pre-exposure. Conditioning to saccharin-containing 0.1% CMC-H induced aversion to saccharin-free 0.1% CMC-H (*P* = 0.0059, unpaired two-tailed Student’s *t* test; Fig. 5a). Conversely, conditioning to 0.1% CMC-H did not induce aversion to saccharin-containing 0.1% CMC-H (Fig. 5b). Since the contradiction would be due to an increased demand for saccharin-containing 0.1% CMC-H by pre-exposure, we performed the experiment without pre-exposure in the next experiment. As expected, conditioning to 0.1% CMC-H induced aversion to saccharin-containing 0.1% CMC-H in the experimental condition without pre-exposure (*P* = 0.0141, unpaired two-tailed Student’s *t* test; Fig. 5c). In contrast to 0.1% CMC-H, conditioning to the more viscous solution, 1% CMC-H, induced aversion to saccharin-containing 1% CMC-H even after short-term pre-exposure to saccharin-containing 1% CMC-H (*P* = 0.0305, unpaired two-tailed Student’s *t* test; Fig. 5d).

### Discrimination of viscosity with different types of thickeners

**Figure 6:**
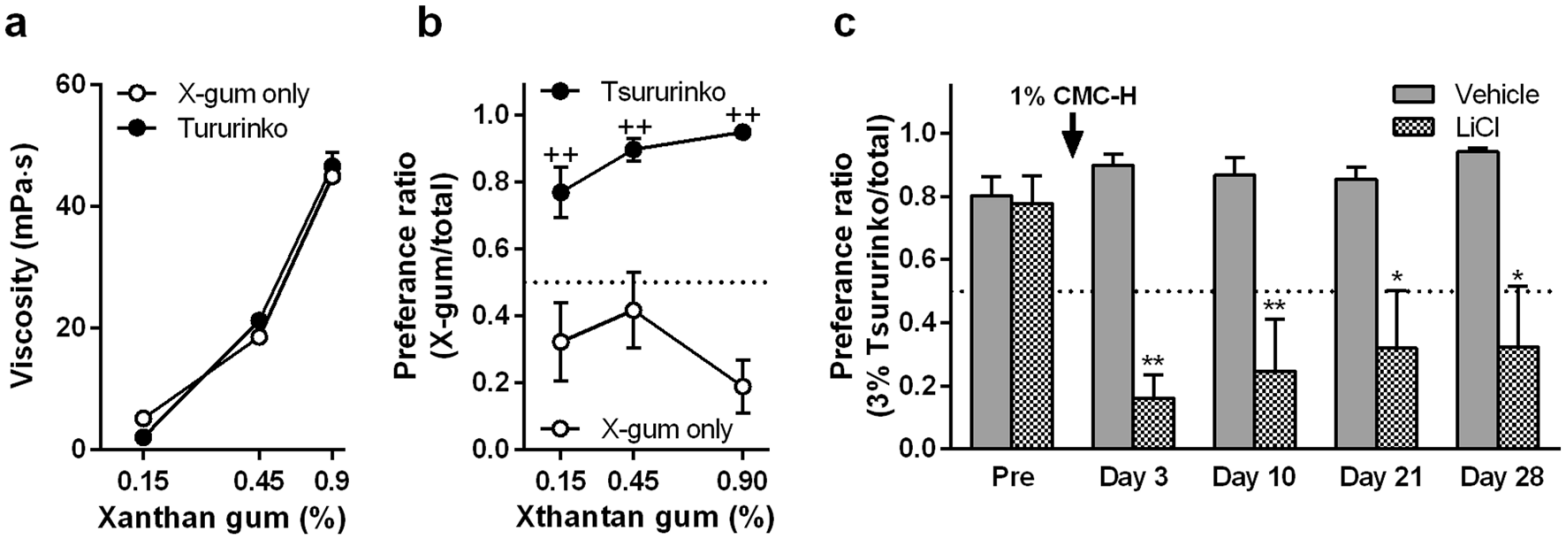
Viscosity discrimination for another type of viscous fluid. Mean ± standard error of the mean. (**a**) Mean viscosities of xanthan gum (X-gum)–containing fluids (3 measurements). The x-axis is a logarithmic scale. (**b**) Preference ratios for X-gum–containing fluids compared to water. (each point, n = 5). ^++^, *P* < 0.01 against fluid thickened only with X-gum in Sidak’s post hoc test following two-way ANOVA. (**c**) Following a short pre-exposure to 3% Tsururinko®-containing fluid, preference ratio in water and 3% Tsururinko®-containing fluid after lithium chloride injection following ingestion of 1% high-viscosity carboxymethyl cellulose (CMC)-containing fluids (CMC-H) as conditioned stimulus (each group, n = 5). * and **, *P* < 0.05 and 0.01, respectively, against vehicle on the same tested day in unpaired two-tailed Student’s *t* test.

Finally, we explored viscosity perception in rats using another type of thickener, xanthan gum, which has a different type of physical property; CMC-containing fluids show Newtonian dynamics (viscosity maintains a constant value regardless of shear rates), whereas xanthan gum–containing fluids show non-Newtonian dynamics (viscosity is decreased when shear rates increase). In our used viscometer that set a constant share rate, the viscosities of xanthan gum at 0.15–0.9% increased in a dose-dependent manner (Fig. 6a). In two-bottle preference tests of xanthan gum vs. plain water, the preference for xanthan gum was comparable to that for water, although the preference for 0.9% xanthan gum was relatively low (no significant difference in one-way ANOVA; Fig. 6b). The commercial thickener Tsururinko®, which contains 30% xanthan gum, showed similar viscosity to xanthan gum at 0.5, 1.5 and 3% (converted to 0.15, 0.45 and 0.9% in xanthan gum) (Fig. 6a). The preference ratios for fluids thickened with Tsururinko® were significantly higher than those for fluids thickened only with xanthan gum (*P* = 0.0023, *P* = 0.0011, and *P* < 0.0001 for 0.15%, 0.45, and 0.9% in xanthan gum, respectively, in Sidak’s post hoc test following two-way ANOVA; Fig. 6b). This result was inconsistent with our previous human study, which reported that Tsururinko® reduced the palatability of water in a dose-dependent manner in human subjects^28^. The difference between rats and humans is probably due to a different preference for other ingredients in Tsururinko®, such as dextrin^29–31^. In condition aversion tests, conditioning to 1% CMC-H induced aversion to 3% Tsururinko® (Fig. 6c). The aversion to 3% Tsururinko® continued for 28 days after LiCl injection (day 3; *P* < 0.0001, day 10; *P* = 0.007, day 21; *P* = 0.0213, day 28; *P* = 0.0121 in unpaired two-tailed Student’s *t* test; Fig. 6c).

## Discussion

The study is carefully designed to minimize taste effects in thickeners in two-bottle preference tests. The distinct forms of the thickeners CMC-L and CMC-H at the same concentrations elicited similar neuronal responses from the taste nerves, indicating that CMC-L and CMC-H are equivalent in taste, qualitatively and quantitatively. Furthermore, by incorporating the weaker LI for viscosity than taste^16–22^, low-viscosity discrimination was detected in the simple experimental setting. The novel approach is easier and simpler than previous approaches using many different types of thickeners^11^ and will be useful to investigate the detailed mechanism of texture perception in mammals.

Rats spontaneously avoided CMC-H above 1% in two-bottle preference tests regardless of saccharin involvement. The results suggest that rats can discriminate high viscosity above 63 mPa·s (1% CMC-H). Such high-viscosity discrimination in rats was reported in an early study^11^. In contrast to the high-viscosity CMC solutions, preferences for below 3% CMC-L and 0.3% CMC-H solutions were equivalent to that for water. Although the chorda tympani nerves responded to 0.1% CMC-L and CMC-H, conditioning with a CS of 0.1% CMC-L failed to induce aversion to 0.1% CMC-H or even 0.1% CMC-L. From these results, the tastes of 0.1% CMC-L and CMC-H seem to be too weak to induce conditioned aversion in rats. Additionally, rats are unable to discriminate the extremely low viscosity of 0.1% CMC-L (1.4 mPa·s) from that of water, which is nearly the same (0.93 mPa·s). Importantly, after the LI to 0.1% CMC taste by long-term pre-exposure to 0.1% CMC-L, rats acquired conditioned aversion to 0.1% CMC-H (Fig. 3d). These results suggest that rats can discriminate the viscosity of 0.1% CMC-H (3.6 mPa·s). With the added viscosity-lowering effect of saliva stimulated by sweet tastes^28^, rats discriminated a viscosity below that of saccharin-containing 0.1% CMC (3.1 mPa·s) but over 1.4 mPa s. Thus, rats can discriminate a considerably low viscosity of 3 mPa·s, similar to that of milk, Worcestershire sauce, or soy sauce.

LI is a phenomenon in which pre-exposure to a stimulus inhibits subsequent acquisition of a conditioned response to that stimulus, as explained by 2 theoretical models; the acquisition model considers that subsequent learning is impaired, but the retrieval model considers that memory expression is impaired. The retrieval model has been supported by recent studies^22^ rather than the acquisition model. In the conditioned taste aversion test, LI to saccharin has been reported to be induced by a single pre-exposure for only 5 min^18,19,21^. However, Polycose, a starch-derived polysaccharide, at 30% (viscosity is 4.0 mPa·s^23^) has been reported to fail to induce LI after a short-term pre-exposure^24–27^. Similarly, the present study demonstrated that short-term pre-exposure to 0.1% CMC-H was not enough to induce LI to the same solution, although long-term pre-exposure established LI. The weaker magnitude of LI to viscosity than taste may be due to the difference in novelty between taste and food textures because food textures are experienced during meals, different from sweet taste, etc. However, such low novelty of texture leads to emphasizing LI efficacy. Hence, the difference is denied because of the weaker LI of viscosity. Another possibility is that differences in the ascending pathway between taste and tactile sensations may affect memory retention in latent learning or retrieval processes in novel learning.

Generalization is a phenomenon in which a behavior that has been established to one stimulus is also elicited by other stimuli and explained in terms of similarity between stimuli^32,33^. In the present study, the conditioned aversion to high viscosity (60 mPa·s) was generalized to low viscosity (3.6 mPa·s). Rats spontaneously avoided CMC-H over 1%, indicating that rats identified a difference in viscosity of CMC-H at 0.1% and 1%. Hence, the generalization seems to be induced by recognition of the physical similarity of viscosity in 0.1% and 1% CMC-H. Additionally, the conditioned aversion to 0.1 and 1% CMC-H was generalized to saccharin-containing 0.1 and 1% CMC-H, respectively. Furthermore, conditioned aversion to Newtonian solution (1% CMC-H) was generalized to non-Newtonian solution (3% Tsururinko®, 45 mPa·s). In these generalizations, rats may recognize the physical similarity of different viscous fluids that have different tastes or different characteristics.

In general, various viscous foods, such as honey and sauce, contain tastes. In humans, viscosity and tastes are perceived independently, although some studies have reported that the addition of a taste to viscous fluids changes the oral perception of viscosity^34^. Oral tactile and taste information are transmitted to the cerebral cortex through adjacent neural pathways. Oral tactile information is transmitted to the somatosensory cortex, insula, amygdala and orbitofrontal cortex through the nucleus ventralis posteromedialis thalami^35^, while taste information is transmitted to the insula, amygdala and hypothalamus through the solitary tract and parabrachial nucleus^36–39^. The difference may be related to the difference in LI intensity. Since several studies have reported that part of the solitary tract and the parabrachial nucleus might relay both taste and mechanical information to the orbitofrontal cortex^40^, taste and texture perception would be related to each other. Thus, integrated analysis of taste and texture perceptions is required in the future.

In conclusion, rats can discriminate considerably low viscosity independent of taste. By combining behavioral, physiological, and molecular experiments, our approach may be able to investigate the molecular and neurological mechanisms of texture sensation and perception.

## Data Availability

The datasets generated during and analyzed during the current study are available from the corresponding author on reasonable request.
